# Collaborative Gaze Channelling for Improved Cooperation During Robotic Assisted Surgery

**DOI:** 10.1007/s10439-012-0578-4

**Published:** 2012-05-12

**Authors:** Ka-Wai Kwok, Loi-Wah Sun, George P. Mylonas, David R. C. James, Felipe Orihuela-Espina, Guang-Zhong Yang

**Affiliations:** 1 Hamlyn Centre for Robotic Surgery, Imperial College London, London, SW7 2AZ UK; 2 Department of Computing, Hamlyn Centre for Robotic Surgery, Imperial College London, Bessemer Building, B510 Level 5, South Kensington Campus, London, SW7 2BZ UK

**Keywords:** Robotic surgery, Human–robot interface, Eye tracking, Perceptual docking, Collaborative surgical task

## Abstract

The use of multiple robots for performing complex tasks is becoming a common practice for many robot applications. When different operators are involved, effective cooperation with anticipated manoeuvres is important for seamless, synergistic control of all the end-effectors. In this paper, the concept of Collaborative Gaze Channelling (CGC) is presented for improved control of surgical robots for a shared task. Through eye tracking, the fixations of each operator are monitored and presented in a shared surgical workspace. CGC permits remote or physically separated collaborators to share their intention by visualising the eye gaze of their counterparts, and thus recovers, to a certain extent, the information of mutual intent that we rely upon in a *vis-à-vis* working setting. In this study, the efficiency of surgical manipulation with and without CGC for controlling a pair of bimanual surgical robots is evaluated by analysing the level of coordination of two independent operators. Fitts’ law is used to compare the quality of movement with or without CGC. A total of 40 subjects have been recruited for this study and the results show that the proposed CGC framework exhibits significant improvement (*p* < 0.05) on all the motion indices used for quality assessment. This study demonstrates that visual guidance is an implicit yet effective way of communication during collaborative tasks for robotic surgery. Detailed experimental validation results demonstrate the potential clinical value of the proposed CGC framework.

## Introduction

In the last two decades, minimally invasive surgery (MIS) has become a matured surgical discipline that reduces scarring, blood loss, and patient recovery time. The introduction of surgical robots has further enhanced manual dexterity, precision, and ergonomic control of MIS. Master–slave systems such as the da Vinci^®^ robot, allow the performance of remote procedures by having the surgeon operating through a surgical console with magnified 3D vision combined with motion scaling and seamless control of the EndoWrists^®^ inside the patient. Remote collaboration through a common robotic platform has been the main motivation of many early attempts of tele-operation (e.g. Marescaux *et al*.^11^). Commercial systems such as the da Vinci Si now offer the possibility of two surgeons operating collectively through two separate master consoles to control multiple surgical instruments.

Collaborative surgery has several advantages compared to the conventional master–slave approach since it allows several expert surgeons with complementing skills to perform a surgical procedure simultaneously. It permits the sharing of expertise and knowledge whilst enabling each surgeon to manage or lead different parts of the procedure. This brings the current robotic surgery closer to the traditional workflow and is particularly useful for complex tissue manipulation tasks that are beyond the capability of bimanual control of a single surgeon. The platform also permits remote mentoring or assistance, with which the remote expert surgeon can take over a part of the procedure when it is deemed to be too difficult to the local surgeon or trainee.

Although collaborative robotic surgery represents an attractive approach, it suffers from a number of difficulties mainly due to the physical divide of the operators, since this removes many of the perceptual cues we rely upon for gauging the anticipated actions of their collaborators. For the current systems, although verbal exchange is always maintained, shared control of the surgical instruments over the same surgical scene still leads to confusion and hinders efficient and safe interaction between the collaborating surgeons. These can lead to instrument collision and inadvertent tissue damage, raising significant patient safety concerns. During a complex collaborative surgical procedure, it is necessary that collaborators can share their intention without explicit verbal exchange of words. It is because verbal guidance can be cumbersome and misleading when manipulating along complex anatomical pathways. During verbal interaction, clear understanding of the meaning of certain words and phrases may also require contextual information and often involves gestures or other communication media. During interaction between surgeons, linguistic disambiguation is often performed with simple referencing gestures. Pointing is a simple and direct way of referencing. For example, Clark^3^ explained the phenomenon that pointing at an object in space, leads the conversing participants to shift attention towards the object, with a consequent disambiguation of context and an economy of words. However, pointing for referencing is not always practical when both interlocutors’ hands are operating the instruments at the same time. It also becomes impossible when the instruments are moving and the scene is dynamically changing. Consequently, time is wasted for correcting misunderstandings between interlocutors. Naturally, an implicit way of communicating intention is the key to the success of seamless collaboration.

Most recently published work uses eye gaze as a means of more effective human–machine interaction and for facilitating hand-eye coordination.^12^ We have proposed previously the concept of *perceptual docking* for robotic control, with which eye-tracking has been used as one of the key perceptual cues for robotic control.^17^ The use of eye-tracking, however, has a long research history. One of the common uses of eye-tracking is for assisting disabled people. In Hutchinson *et al*.,^8^ gaze is used to type on a keyboard that is displayed on a screen in order to select other functionalities, enabling them to interact and control their environment. Real-time eye tracking and saccadic eye movements have also been used for robotic control and improving visual-motor coordination.^12^ Compared to the use of other input modalities such as mice, touch screens, and pointers, eye gaze is able to implicitly carry information on the focus of the user’s attention at a specific point in time.^16^ In fact, eye gaze is a fundamental cue we rely upon for face-to-face communication. Effective communication is naturally intertwined with eye gaze. For instance, speakers would normally demonstrate their focus by looking. Looking away or avoiding direct eye contact may reflect hesitation, embarrassment, or shyness. Griffin and Bock^6^ showed that when speakers are asked to describe a simple scene, they tend to fixate on the objects in the order in which they are mentioned and roughly 800–1000 ms before naming them. Richardson and Dale^13^ demonstrated the close coupling occurring between speakers’ and listeners’ eye movements and its relationship to discourse comprehension over the same visual scene. Speakers’ and listeners’ eye movements were tracked throughout during the speech. The eye movements of speakers and listeners were showed to be linked. How closely listeners were following a speaker’s gaze predicted how well they would answer comprehension questions. The most relevant work recently proposed under the paradigm of Computer Supported Cooperative Work (CSCW) was conducted by Jermann *et al*.,^9^ who attempted to understand high-level cognitive behaviour based on the dual gaze patterns in a collaborative task. All these suggest that gaze is not just a perceptual channel, but more importantly, a communicative one.

In order to facilitate or enhance collaboration in a shared multi-robot surgical environment, it would be desirable to reveal the visual attention of the collaborating counterparts. The study presented here aims at demonstrating how the concept of Collaborative Gaze Channelling (CGC) can achieve this goal. In this paper, the efficiency of surgical manipulation with and without CGC for controlling a pair of bimanual surgical robots is evaluated by analysing the level of coordination of two independent operators. Fitts’ law is used to compare the quality of instrument movement with or without CGC in a study group of 40 subjects. The results show that CGC can enhance cooperation amongst surgeons on a master/assistant paradigm by improving speed, accuracy, and reliability during a collaborative task.

## Materials and Methods

**Figure 1:**
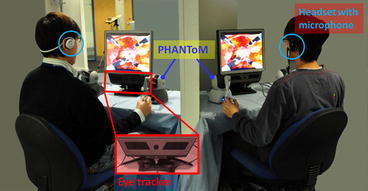
Illustration of the experimental setup for simulated collaborative manipulation: two workstations are used, each one equipped with remote eye tracking capability. For the use of verbal guidance, operators communicated through their headsets

For studying the role of eye-gaze and the effectiveness of CGC, a remote collaborative surgical environment has been developed. This involves the use of two mirrored remote visualisation and control stations. The two stations (Fig. 1) are separated in such a way that is similar to the current multi-console operating environment of robotic surgery. The screens are used to display identical views of the shared surgical environment to the two collaborators. For capturing visual attention, each surgical station is equipped with a remote eye tracker that allows determining the fixation point of the operating collaborators within the shared surgical scene.

### Surgical Console

For capturing visual attention, each of the two surgical consoles is equipped with a video-oculography eye tracker. Due to the geometry of the eye, the corneal reflection vector can be uniquely assigned to a gaze direction. After a brief calibration routine, such reflection vectors can be mapped into unique fixation points onto the stimulus screen in use. Figure 1 shows the eye-tracking device used in this study, as a stand-alone remote eye tracker positioned under the screen. For this study, a Tobii x50 (Tobii Technologies AB, Sweden) eye tracker is used. The system is able to track fixation points at 50 Hz with an accuracy of 0.5° and drift <1 degree across the work plane. It allows for a certain amount of head movement within a working volume with dimensions of 30 × 16 × 20 cm^3^ (*W* × *H* × *D*).

**Figure 2:**
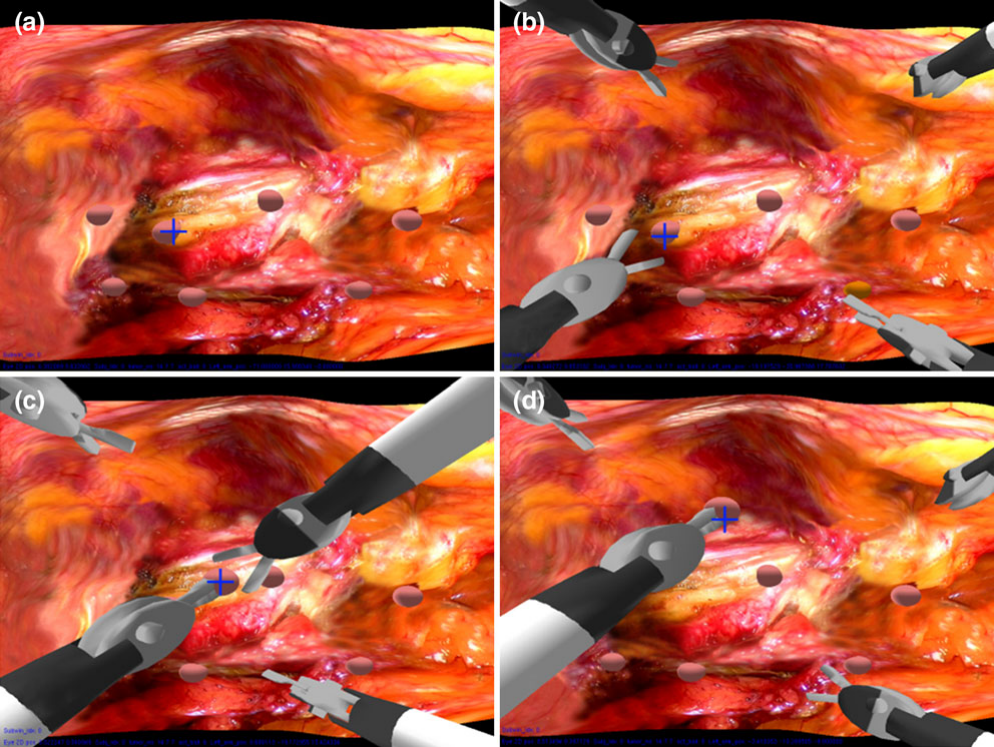
A synthetic surgical scene rendered with the 3D prostate tissue is adopted to perform nodule extraction. The blue cross displayed on the screen represents the eye gaze position of the master. (a–d) An image sequence showing the main steps involved in extracting a single nodule during the experiment: a particular nodule being identified, the assistant extracts it by using the instrument on his/her left or right and passes to the instrument operated by the master, and the nodule is then eliminated

For collaboration within the shared surgical scene, both surgical stations provide visualisation on 17-inch LCD monitors at a resolution of 1024 × 768 pixel. It is worth noting that the resolution of the eye tracker is determined by the visual angle. The current screen resolution used already exceeds the achievable per-pixel accuracy of the eye tracker. For the experimental setting used for this study, approximately ten effective pixels are covered by a visual angle of 0.5°, which is the average accuracy of the Tobii eye tracker used. Control of the two laparoscopic instruments, which are linked with two virtual robotic arms, is achieved using two PHANToM Omni haptic devices from SensAble Technologies (MA). Figure 2 shows a snapshot of the shared synthetic scene used for the study. The procedure simulates a simple tissue manipulation task during MIS that involves extracting a small nodule and passing it on to the collaborator. The small spheres represent nodules that can be picked up and removed using the surgical instruments. The upper two instruments can be controlled by the manipulators on one console while the other two by those on the other console. In this way, two users can collaborate within the shared environment. The eye trackers capture an operator’s fixation point in real time and present it to their collaborator’s screen by means of a blue cross. Under this setup, each of the two collaborators is able to see a visual representation of their counterpart’s visual point of attention. Two identical consoles were used for the two participants; one designated as the master and the other as the assistant during the experiment. The participants were seated in front of their console and although located in a same room they are visually separated by a partition to avoid face-to-face interaction (Fig. 1). Care was taken so ambient conditions are consistent throughout the experiment. Having finished an eye tracking calibration routine, they can start to control each designated pair of virtual laparoscopic tools through the PHANToM devices on each console. As shown in Fig. 2, the two instruments shown on the bottom of the synthetic scene are operated by the assistant and the two on the top are operated by the master.

### Experimental Design

For the simulated surgical task for nodule extraction, a total of seven nodules are located on a 3D tissue surface rendered with realistic prostate tissue texture (Fig. 2). To simplify the task, the nodules can be extracted from the tissue surface by any of the assistant’s surgical tools. After a nodule is being picked up, it can be eliminated from the scene by passing it over to one of the master’s instruments. The study involves extraction of all seven nodules and the order in which nodules are to be removed is predetermined and only shown to the master who is responsible to communicate its location to the assistant.

Verbal guidance is used for the control experiment. Having standardised the set, the master is only allowed to describe the nodule locations using a limited set of instructions. These instructions referring to the location of the target nodule, such as *left/right upper corner* or *centre*, or further *left/right/up/down*, express instantaneous indication of the direction respect to the *left/right* instrument tip. Confirmations such as *yes* or *no* somehow are required. For the fair evaluation, the communication is only in one way from the master to the assistant. Verbal dialogue for disambiguation is allowed when necessary and the master can also repeat the instructions. Guidance in the CGC case is implied by the position of the master’s fixation point as this is relayed and visualised on the assistant’s screen. Similarly, in parallel, the master is able to see the assistant’s fixation point on their monitor. The typical sequence of events for both the vocal and CGC guidance tasks is outlined in Table 1.

**Table 1:** The typical sequence of events for a single-nodule manipulation task

Step 1	A particular nodule is identified to the master who is responsible to communicate its location to the assistant
Step 2	The assistant can use bimanual control to grasp the indicated target with the gripper of one of the instruments. Depending on the position of the nodule, one instrument may not be able to reach it. In this case, the instrument closer to it has to be used. This step is repeated until the nodule is successfully grasped and removed from the tissue surface
Step 3	The assistant passes the nodule to one of the master’s instruments. The master needs to pick up the nodule by operating the gripper at the end the instrument
Step 4	Successful grasping of the nodule by the master, automatically removes it from the scene
Step 5	All above steps are repeated until the pre-allocated task time lapses

The above procedure is also illustrated in Fig. 2. For consistency, the task duration was constrained to 30 s and each task was repeated five times by each subject allowing rest time in between. During the rest periods, the subjects maintain their position. The order between the control and the CGC sets was randomised. The total completion time of an experiment was 16 min, consisting of 3 min for briefing, 3 min for warm-up practice, and 5 min for all tasks (five times vocal and five times CGC). All subjects were allowed to familiarise themselves with the control of the haptic devices before data collection took place. Before the experiment, informed consent was given to all subjects and each has been asked to sign a consent form. A total of 40 subjects (36 male plus 4 female) were recruited to participate in the experiment. It includes 14 biomedical engineering students, 13 biomedical engineering research fellows, and 13 surgeons. The average age is 28.5 ± 4. To ensure consistency, all 40 subjects took the role of surgical assistant and their performance was evaluated through the measurement of indicators which are explained in detail in the following sections.

### Eye Tracking

For analysing eye tracking data, time integral of gaze displacement is defined, where gaze displacement represents the spatial offset between the master’s and assistant’s fixations at the moment when the master requests removal of a specific nodule. Perfect gaze convergence between the master and the assistant occur when both fixation points are collocated at the point where the master was intending to attract the assistant’s attention.

The *Gaze Latency* is defined as the amount of time between a nodule removal request and the eventual gaze convergence. In determining *gaze latency*, two conditions should be satisfied when the respective regions-of-interest (ROIs) are fixated upon: (1) The convergence tolerance should be within the average size of the nodule on the display (13 mm in this study); (2) The assistant’s gaze should dwell for more than 300 ms within the convergence tolerance. This is the time from the beginning of two gazes’ convergence until reaching the dwell time threshold.

*Gaze Convergence* is an integral of the gaze displacement between the master and the assistant over the time it takes for the two to merge within the defined convergence tolerance. It represents the actual visual search that took place. In addition to gaze convergence, the correlation *R*-value needs to be examined between the master and the assistant fixations. For this, scatter plots of the assistant’s vs. the master’s fixation coordinates need to be generated and the correlation to be calculated through linear regression. These three parameters are essential performance indices for revealing the assistant’s performance and understanding of the task.

**Figure 3:**
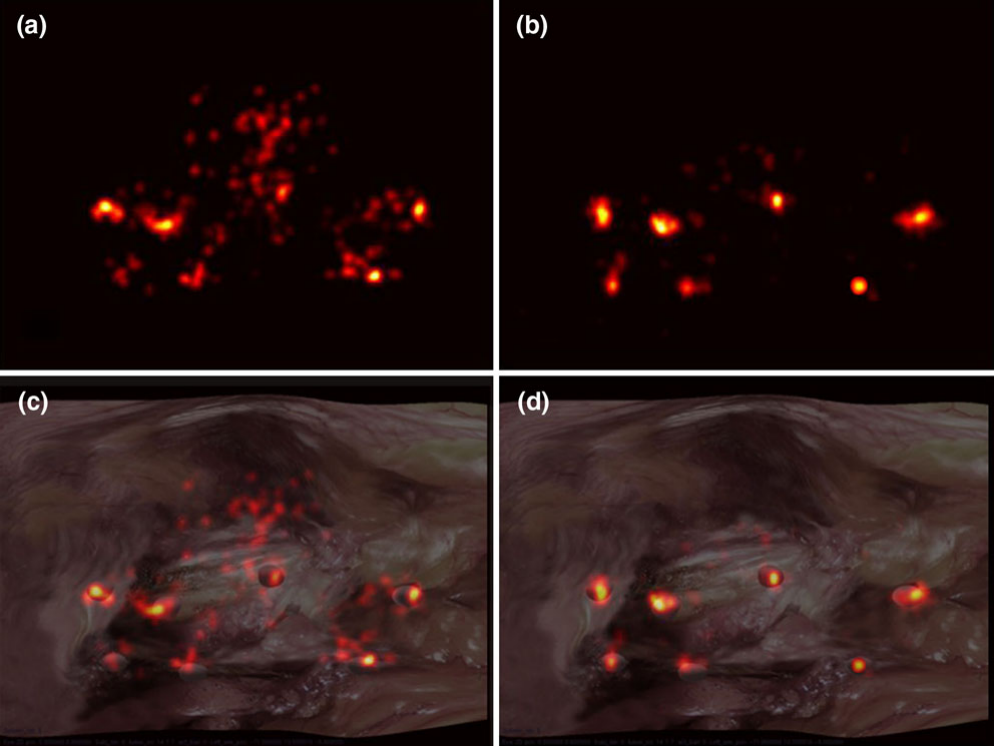
Example hotspot representation showing an assistant’s fixations during one control task (a) and one CGC task (b), also superimposed over the synthetic surgical scene (c) and (d)

The eye tracker operates and measures gaze positions at a sampling rate of 50 Hz. Although inadvertent or involuntary gazes do happen occasionally (e.g. due to distraction), the gaze is only used to convey the attention cues of the collaborating surgeon. It is therefore important to show this information as is to the collaborating partner. Only basic filtering (median) is performed to reduce noise due to the eye tracking hardware and ocular tremor. In order to visualise the recorded data intuitively, instead of plotting every discrete gaze position that makes the results unclear, fixation clustering was applied and presented as a hotspot map. Fixation clustering is achieved through convolution of the plotted fixations over the image space with a spectrum mask of adjustable diameter and weights. The convolution mask is formed by a Gaussian kernel with dimension 40 × 40 pixels out of the full screen resolution of 1024 × 768. It is set to be smaller than the size of a nodule in 2D and to be roughly equal to an area subtending an average fovea with 1° of visual angle. The standard deviation of the kernel is set to 6.5 and the maximum weight applied at the centre of the mask. When a fixation is plotted once at each sample time, the colour intensity at the centre of the mask will be increased by 1.8 units. If the fixation dwells at the same point for more than 2.83 s, a bright spot with maximum intensity of 255 will be observed. All these parameters introduced for the kernel are determined with the aim of having sharp and distinguishable fixation spots on the map. Hotspot maps, like the one shown in Figs. 3a and 3b, represent an intuitive visualisation method where higher fixation concentration areas, over a certain time period, are shown as brighter coloured spots.

### Instrument Motion Analysis

For analysing instrument motion, the total distance travelled by the instrument was measured. This is for evaluating performance efficiency during the task. In the context of this study, we use the accumulated distance travelled which is defined as the total distance travelled by the assistant’s instrument from the moment when the desired nodule was verbally or visually indicated by the master until it has been picked up from the tissue surface. The system recorded the accumulated distances travelled by both the left and right instruments held by the assistant. The time required to complete the task, the instrument tip distance travelled and the speed were also measured. Subject specific analysis was used to compare task performance between vocal and CGC guidance. The hypothesis to be evaluated is whether the application of CGC can enhance the cooperation in terms of speed, accuracy, and reliability.

In the current study, Fitts’ law is applied to measure and compare the assistant’s response with and without CGC. This law was first developed by Fitts as an empirical model to analyse speed and accuracy trade-off of human muscle movement.^4^ The early experiments were related to pointing and targeting movements to estimate a worker’s underlying efficiency. Subsequent studies^1^ have successfully applied Fitts’ law in a variety of conditions including eye gaze.^10^ It is also often used as a model for pointing actions in user interfaces or human–computer interaction. Based on the well-known Shannon–Hartley theorem,^15^ Fitts’ law can be formulated as:

1 $$ MT = a + b\left( {ID} \right)\quad \text{s.t.}\quad ID = \log_{2} \frac{2A}{W} $$

where *MT* denotes the moving time required to hit the target. The *ID* is a logarithmic function of the spatial relative error varied by the parameters *W* and *A*, respectively, which are the width of the target nodule and the travelled distance of the tool tip when gripping the nodule. This distance is recorded from the point when the previous nodule is passed over to the master’s instrument to the other point when the target nodule is gripped. For this study, each task was repeated five times. In order to standardise the *ID* calculation, such recording is skipped for the first nodule gripping in each task. In Eq. (1), *a* and *b* are empirical constants which can be determined experimentally by fitting a straight line to measured data. Fitts’ law predicts that the time *MT* to pick up the target (nodule) depends on its width *W* (diameter of the nodule, *ϕ* = 13 mm) and its distance *A* to the cursor (in this case the instrument tool tip). In order for the nodule to be picked up, the tool tip must fall within $\pm \frac{W}{2}$ of the nodule’s centre.

The logarithmic term is defined as the index of difficulty *ID* of a target. In addition to the index of difficulty, Fitts also defined the Index of Performance (*IP*). The *IP*, is expressed in bits/time and can be used to characterise how quickly pointing can be done, independently of the involved target characteristics. Measuring the *IP* of different devices or systems allows their comparison with respect to their pointing capability. There are two different representations of *IP* in the literature. One of them is $IP_{1} = \frac{1}{b}$, which was defined to compare performance on a mouse selection task on a screen.^1^ However, based on Fitts’ theorem, the effect of *a* is ignored and it is only valid under ideal circumstances such as no learning curve effect taking place, which is considered as a restriction of this representation. An alternative way of representing the *IP* 18 is $IP_{2} = \frac{{ID_{\text{average}} }}{{MT_{\text{average}} }}$, where *ID*_average_ is the average of the index of difficulty for a target and *MT*_average_ is the average of the moving time taken to hit this target. Although we endeavour to minimise the learning effect by using randomised control trials, both indices were calculated in order to present the performance with and without the consideration of the learning effect in this study.

## Results

For all 40 subjects studied, the following figures summarise the key findings. Figures 3a and 3b show a typical hotspot fixation plot of one of the subjects with verbal and CGC guidance respectively. The same time scale was used in both cases for colour mapping. In these figures, the brighter the colour, the longer the duration of the fixations on that particular region. Figures 3c and 3d illustrate the aforementioned hotspot plots superimposed onto the surgical view. It is evident that in the CGC case, the majority of the fixations are well clustered around the nodules. This signifies efficient visual search patterns. In the case of verbal guidance, the fixations are more widely distributed, largely due to the confusion of the subject with their fixations darting around the visual scene in an attempt to determine the requested target that the master is trying to communicate through verbal instructions.

**Figure 4:**
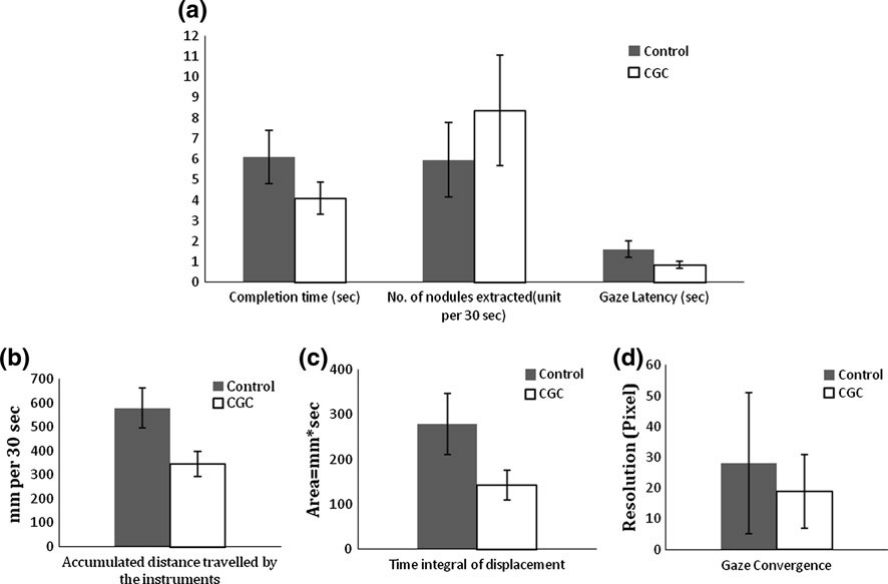
(a–d) Six performance indicators for the control (in gray) and CGC (in white) experiment among 40 subjects: (1) completion time; (2) number of nodules extracted; (3) gaze latency; (4) accumulated distance travelled by the instruments; (5) time integral of gaze displacement; and (6) gaze convergence

In order to present these results quantitatively, Figs. 4a–4d summarise the mean and standard deviation for the six performance indicators, namely completion time, number of nodules extracted, accumulated distance travelled by the instruments, gaze latency, time integral of gaze displacement and gaze convergence among all the 40 subjects studied. In all six cases, the improvement offered by CGC is evident. Figure 4a summarises the average time required by all subjects to complete a single tissue extraction for the control and CGC tasks, plotted along with the respective gaze latency. The gaze latency is also indicated as a proportion of the completion time, in percentage of 20.6 and 26.3%, respectively, with and without CGC. For instance, 50% improvement with smaller standard deviation is evident for gaze latency. Again from Figs. 4b–4d, CGC guidance considerably improves efficiency of the collaborative task.

**Figure 5:**
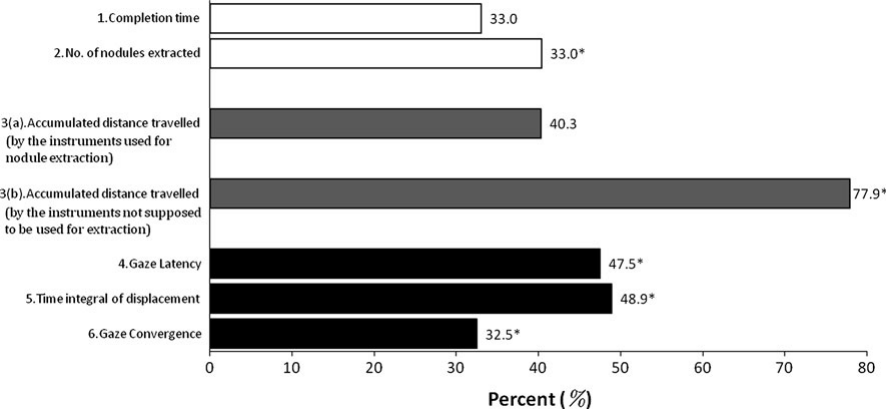
Summary of the improvement with all six indicators classified in three main categories, namely: outcome (in white), instrument (in gray), and gaze (in black). The six indicators are (1) completion time; (2) number of nodules extracted; (3a and 3b) accumulated distance travelled by the instruments including both the one that successfully extracted the nodule and the one not used for the extraction; (4) gaze latency; (5) time integral of gaze displacement; and (6) gaze convergence in percentage change (* indicates significant difference, *p* < 0.05)

Figure 5 shows the overall improvement for the CGC task compared to verbal guidance, evaluated by using the six performance indicators for all the subjects studied. The percentage improvement over the control experiment on all performance indices ranges from 33 to 49% (ranging of *p* < 0.05 in between [0.004, 0.01]). More specifically, the number of nodules extracted is increased by more than 40% with CGC, the task completion time is shortened by 33%, the total distances travelled by the active instrument is shorter by more than 40%. Table 2 summarises the six performance indicators for the 40 subjects studied and Table 3 summarises the paired difference of these performance indices between the two Guidance methods. All performance indicators were found to be improved in the tasks using CGC. For the number of extracted nodules, more nodules were extracted by the assistant with CGC. Smaller values of indicators, excluding the number of nodules, were found in the tasks using CGC. All differences among six indicators were significant by the *p*-values (*p* < 0.05) obtained on the paired test (Table 3).

**Table 2:** Summary of values of six performance indicators for 40 subjects between the two Guidance methods

	Control		CGC	
	Mean	SD	Mean	SD
Completion time (s)	6.1	1.3	4.1	0.8
No. of nodules extracted (unit per 30 s)	6.0	1.8	8.4	2.7
Accumulated distance travelled by the instrument (mm per 30 s)	578.4	84.4	345.3	52.0
Gaze latency (s)	1.6	0.4	0.8	0.2
Time integral of displacement (mm s)	278.4	68.5	142.4	33.7
Gaze convergence (pixel)	28.1	22.9	18.9	12.0

**Table 3:** Paired differences between the two Guidance methods

	95% confidence interval				
	Mean	SD	Lower	Upper	*p*-value
Completion time (s)	2.01	1.13	1.65	2.37	0.000*
No. of nodules extracted (unit per 30 s)	−2.4	2.02	−3.05	−1.76	0.000*
Accumulated distance travelled by the instrument (mm per 30 s)	233.2	92.6	203.5	262.8	0.004*
Gaze latency (s)	0.76	0.36	0.65	0.88	0.000*
Time integral of displacement (mm s)	136	64.44	115.34	156.57	0.000*
Gaze convergence (pixel)	11.76	24.3	4.0	19.5	0.01*

Paired differences = difference of methods using the control experiment minus CGC

*Significant at *a* = 0.05 (2-tailed)

**Figure 6:**
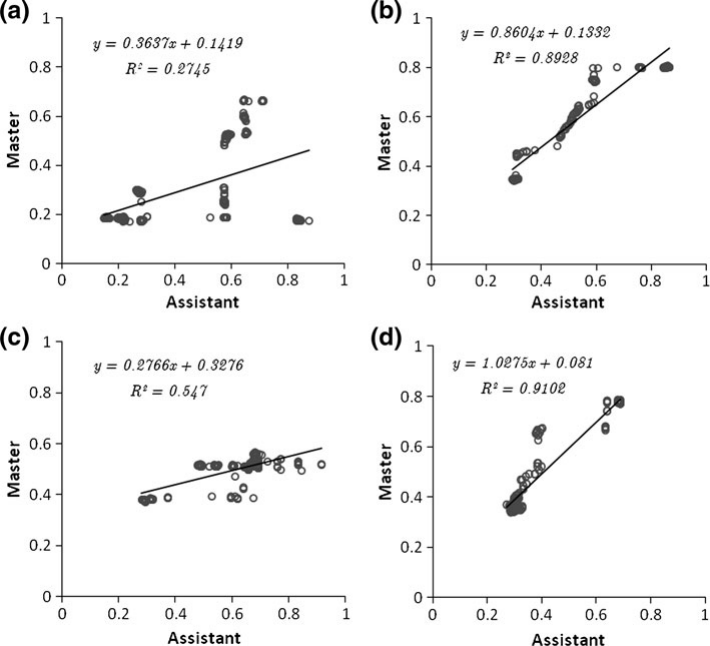
The regression of master’s gaze against assistant’s in one of the subject tests. Line is fitted among the data and *R*-value is then calculated. The graphs illustrate the gaze movement in horizontal in the control (a) and CGC (b) experiment. The graphs illustrate the gaze movement in vertical in the control (c) and CGC (d) experiment

Figure 6 shows an example regression plot between the assistant’s and the master’s fixations. In this figure, the left column corresponds to the control task and the right corresponds to the CGC enabled task. The top row of the figure shows the fixations along the horizontal *x*-axis on the screen coordinates whereas the bottom row shows the fixations along the vertical *y*-axis. Significant differences in pattern distribution are evident—with CGC, the data sets are clustered relatively densely along the regression line. Stronger correlation is shown between those two gaze data sets and the corresponding *R* value is closer to 1. Compared to verbal guidance, significantly fewer outliers can be observed with CGC. The same trend can be observed for all the 40 subjects studied. Table 4 summarises the corresponding regression values (mean, standard deviation, and range) for all the subjects studied. For verbal guidance, *R*-values on the horizontal and the vertical axis are ranging from 0.11 to 0.68 and 0.11 to 0.84 respectively. During CGC, these are ranging from 0.31 to 0.95 and 0.32 to 0.95 for horizontal and vertical respectively. The mean and standard deviation for *R* are also presented and demonstrate significantly higher correlation for the CGC case.

**Table 4:** Summary of regression between master’s and assistant’s gaze and *IP* for 40 subjects between the two Guidance methods

*R*-value	Control		CGC			Paired *t*-test		
	Mean	SD	Range	Mean	SD	Range	Diff	*p*-value
Fixations in horizontal axis	0.33	0.18	0.57	0.65	0.16	0.63	0.32	0.000*
Fixations in vertical axis	0.5	0.23	0.74	0.65	0.15	0.63	0.15	0.002*

Index	Mean	SD	Mean	SD
IP_1_	1.88	0.65	2.6	1.53
IP_2_	0.51	0.24	1.11	0.41

Diff = difference of methods using CGC minus the control experiment

*Significant at *a* = 0.05 (2-tailed)

**Figure 7:**
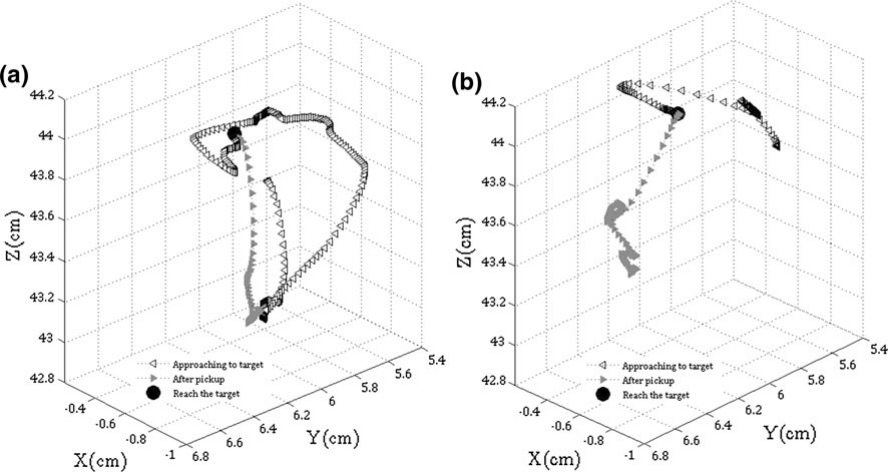
Trajectories of an instrument tip maneuvered by an assistant in 3D space during a single nodule extraction task in the control (a) and CGC (b) experiment

**Figure 8:**
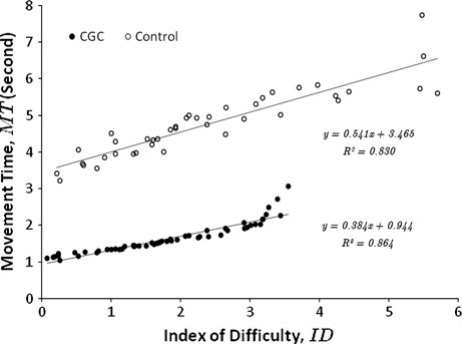
Plots of movement time against index of difficulty using Fitts’s law for assistant in a single subject test

Figure 7 shows typical trajectories of the instrument tip of one of the assistants studied with verbal (a) and CGC (b) guidance. In the nodule extraction task, after the target nodule is recognised by the assistant, instrument trajectories demonstrate very different patterns between the control and the CGC experiment. It is observed that the instrument manipulation is relatively smoother, shorter in length, and more directly approaching the target during CGC, compared to the control experiment. For detailed instrument motion analysis, Fig. 8 illustrates the movement time to hit the target against its index of difficulty for both the vocal and CGC cases. This particular subject successfully extracted 40 nodules during the experiment. Least-squares fitting was used to generate the regression lines and the corresponding values of *a* and *b* in Fitts’ law were calculated in both cases. The values of *b* for the control and CGC cases are 0.54 and 0.38 respectively. Smaller value of *b* implies that the task performance movement time is less affected by the difficulty level. Less movement time was required for performing the CGC tasks compared to the same level of difficulty during the control task. In both cases the correlations are quite strong with the *R*-values around 0.8. It can be observed that some data samples deviate from the linear regression when their *ID* is high. It is because the subject occasionally blocked the view of the target nodule by their instruments. Although the master could potentially guess the target location and spot the fixation on the instrument, the subject would hesitate in searching the target. Such hesitation causes the value of *A* increased, but also deteriorates the smoothness of the manipulation so that their movement time (*MT*) is too unexpected to follow the regression. Table 4 summarises the indices of performance *IP*_1_ and *IP*_2_ for all subjects. Higher values of *IP*_1_ = 2.6 and *IP*_2_ = 1.11 are shown for the CGC as compared to *IP*_1_ = 1.88 and *IP*_2_ = 0.51 for the control experiment. As the task does not require any specific surgical skill, there is no performance difference found between surgeons and the other 27 subjects.

## Discussion

In this study, we have presented a framework based on eye-tracking for improved collaborative manipulation of surgical robots. To minimise the distraction of motor memory for the surgical manipulation, as well as to intuitively describe the location of complex features in the surgical scene, eye gaze rather than explicit pointing and directing was used in this study to convey the attention cues of the surgeons. This is because during surgery the hands of the surgeons tend to be fully occupied with surgical instruments. This alleviates the need for the surgeon to leave control of the tools. It has been shown that eye gaze is more advantageous in terms of implicitly carrying information on the focus of the surgeon’s attention, compared to the use of other input modalities. Multiple performance indices were introduced to assess the performance of a surgical task. Fixation analysis reveals the subjects underlying visual attention and permits more detailed assessment of factors affecting surgical performance. The use of saccades would be interesting but it can be influenced many compounding factors, which are more difficult to decode. Other indices, such as blink rate, were not measured in this study, which can potentially be incorporated into the current framework as they have shown to be relevant to performance in general surgical procedures.^14^ This work supports our original hypothesis that measurement of visual attention facilitates overall hand-eye coordination during an instructed surgical manipulation task and in a collaborative working environment. It further enhances seamless coordination of the team. There is also a concern of penalising the control task by not allowing verbal instructions such as “extract the right most nodule in the right upper quadrant.” Instructions of this nature are also relevant to other examples: “grasp the gall bladder by Hartmann’s pouch” or “lift the tip of the appendix.” However, this is not necessarily the case in relation to the fine adjustment of performance which is often given in terms of “left/right” and “more lateral/medial.” For the case that a trainee is being guided through laparoscopic suturing, which is a very complex task, precise performance adjustment would need to be given in terms of basic directional instruction. Therefore, this scenario does represent a current practice. Our study demonstrates that gaze is as information being conveyed effectively to the assistant.

Instrument motion analysis is excluded from the point that the nodule is being gripped by the assistant’s instrument. The reason for this exclusion is that the transfer movement is a well-identified task and does not require any further instruction or guidance. The transitions consist of very similar patterns of movement and behaviour with and without the use of CGC. It is also important to note that the same person (expert) acted as a master to assess the collaborative behaviour of all the 40 subjects studied. Although it may be argued that exhaustive pair-wise permutation would be useful to assess the performance of each subject when taking different roles and collaborating with different subjects, this would require 780 (=40 × 39/2) experiments, which is impractical in our laboratory settings. The advantage of having one experienced operator as the master ensures the consistency of the data and it better emulates the real-life situation in a collaborative surgical environment, as it typically involves at least one very experienced surgeon and another assistant or trainee. Further analysis of collaborative behaviour between experienced surgeons would be the next step of our study. Due to the nature of the designed task, it is unsurprising that with the same master, the instrument coordination, and trajectories are contrastingly different between subject pairs—highlighting the idiosyncrasy, as well as the importance of effective communication when performing collaborative tasks. Furthermore, in order to understand the brain behaviour during the collaboration task with or without CGC, functional Near Infrared Spectroscopy (fNIRS) is being used to assess the cortical activity patterns in our laboratory.

In this study, gaze convergence and latency have been considered as a key performance index of task quality. Both indices were significantly improved with the use of CGC. More specifically, gaze convergence has increased by 33% and gaze latency has decreased by almost 48%. These show that the subjects (assistants) tended to be more focused on the surgical target, rather than on the plan ahead. The indices of performance $IP_{1}$ and $IP_{2}$ have also been improved. With the use of Fitts’s law, the difficulty of the task was estimated by determining how much time was required for each of these movements and methods with higher indices of performance are proved to be more efficient. One of the limitations of our study is the use of 2D visualization for the surgical scene, which causes a loss of depth perception. This is a known factor for affecting conventional MIS. With the increasing use of 3D displays, particularly on robotically assisted surgical platforms, it would be useful to assess the performance variation with and without 3D perception and further examine other factors that can be taken into account when using CGC enabled collaborative manipulation. Moreover, the proposed CGC can be combined with other means of directing attention. In a recent study, we have studied the effect of different first language on cooperative performance and the value of using eye gaze as the primary guidance.^2^ We also developed a binocular eye tracking system,^12^ which allows to measure the fixation in 3D and can be integrated into the existing commercial surgical console such as the da Vinci^®^. We envisage that eye-tracking control incorporating CGC can be demonstrated by the use of da Vinci^®^ Si HD^7^ for collaborative surgical procedures. With light-weight HD systems emerging on the market, this may change the visual experience and thus is worth pursuing in future. However, it is worth noting that the window of attention is more of a cognitive process,^5^ compared to gaze which only indirectly reflects one’s attention.

In conclusion, this study demonstrates the feasibility of CGC for robotic surgery. The framework was implemented for a multi-instrument manipulation environment, which is gaining significant interest in recent years with the increasing flexibility and miniaturisation of surgical robots and end-effectors. The results derived from this study suggest that CGC is an effective means of communication during surgery as it is natural and does not deviate attention during task performance. It has been shown to be beneficial for cooperative problem solving by preserving the relative gaze position of the operators and their gaze direction. In this paper, the task we used is simplified to allow a detailed assessment of different collaborative behaviour, it is anticipated that the current framework can be extended to complex surgical tasks that involve the navigation of tortuous anatomical pathways and require the use of multiple imaging, tissue manipulation, and focussed energy delivery.
